# Neuropathic pain develops normally in mice lacking both Na_v_1.7 and Na_v_1.8

**DOI:** 10.1186/1744-8069-1-24

**Published:** 2005-08-22

**Authors:** Mohammed A Nassar, Alessandra Levato, L Caroline Stirling, John N Wood

**Affiliations:** 1 Molecular Nociception Group, and London Pain Consortium, Department of Biology, University College London, Gower Street, WC1E 6BT, London, UK

## Abstract

Two voltage gated sodium channel α-subunits, Na_v_1.7 and Na_v_1.8, are expressed at high levels in nociceptor terminals and have been implicated in the development of inflammatory pain. Mis-expression of voltage-gated sodium channels by damaged sensory neurons has also been implicated in the development of neuropathic pain, but the role of Na_v_1.7 and Na_v_1.8 is uncertain. Here we show that deleting Na_v_1.7 has no effect on the development of neuropathic pain. Double knockouts of both Na_v_1.7 and Na_v_1.8 also develop normal levels of neuropathic pain, despite a lack of inflammatory pain symptoms and altered mechanical and thermal acute pain thresholds. These studies demonstrate that, in contrast to the highly significant role for Na_v_1.7 in determining inflammatory pain thresholds, the development of neuropathic pain does not require the presence of either Na_v_1.7 or Na_v_1.8 alone or in combination.

## Background

Voltage gated sodium channels (VGSC) underlie the electrical excitability of nerve and muscle. VGSCs consist of pore forming α-subunits and auxiliary β-subunits. There are ten cloned α-subunits and 4 β-subunits. The β-subunits modulate the localisation, expression and functional properties of α-subunits [1]. Different α-subunits have distinct electrophysiological and pharmacological properties [2]. The complex pattern of expression of α-subunits may imply special roles for particular subunits in different cell types [3]. Many loss- as well as gain-of-function mutations of α-subunits have been identified in human conditions characterised with epilepsy, seizures, ataxia and increased sensitivity to pain. This suggests that mutations of VGSC in humans are significant factors in aetiology of neuronal diseases [4].

Nociceptors are a subset of sensory neurons that respond to noxious thermal, mechanical and chemical stimuli. Nociceptors express multiple subtypes of α-subunits [3]. Tissue and nerve damage leads to changes in expression and function of α-subunits that in turn can lead to change in the excitability of sensory neurons. Changes in the excitability of sensory neurons are thought to underlie some chronic pain conditions [5,6]. Na_v_1.8 and Na_v_1.7 are two α-subunits that are abundant in nociceptive sensory neurons [3,7,8]. Na_v_1.8 is expressed exclusively in sensory neurons and is not found in the CNS [9]. Functional characterization of Na_v_1.8 positive neurons revealed that more than 85% are nociceptors [8]. Na_v_1.7 is expressed principally in peripheral neurons with very weak expression detected in CNS [10,11]. As there are no subunit specific blockers, gene knockouts in mice have provided insights into the role of individual α-subunits genes in pain [5]. Deletion of the Na_v_1.8 gene [12] and nociceptor-specific knockout of the Na_v_1.7 [13] gene have identified a role for these two α-subunits in setting mechanical and, to a lesser extent, thermal pain thresholds. In addition, behavioral studies have revealed deficits in inflammatory pain models, most dramatically in the nociceptor-specific Na_v_1.7 knockout [12,13]. These findings suggest that these α-subunits could be targets for new anti-inflammatory drugs.

Peripheral nerve injury leads to lowered pain thresholds, enhanced responsiveness and/or ectopic activity in sensory neurons that ultimately leads to hyperalgesia and allodynia [5,6]. Changes in expression of α-subunits of VGSCs have been documented in models of peripheral nerve injury [5,6]. This has lead to the hypothesis that modulation of α-subunits in sensory neurons may underlie the increased neuronal excitability of sensory neurons following peripheral nerve injury. Pharmacological blockade of sodium channel activity has been shown to attenuate ectopic activity [14,15] and reverse hyperalgesia following nerve injury [16]. While the role of Na_v_1.7 in neuropathic pain remains to be investigated, analysis of a Na_v_1.8 knockout mouse indicated that it is not involved in alteration of pain threshold following peripheral nerve injury [17]. This is in contrast to the finding of Lai *et al *who reported that antisense oligonucleotides directed against Na_v_1.8 administered intrathecally completely reverse neuropathic pain behavior [18]. It is possible that this discrepancy could be due to the up-regulation of the Na_v_1.7 channel seen in the Na_v_1.8 knockout mouse [12] which might mask an otherwise important role for Na_v_1.8 in neuropathic pain.

In the present study we investigated the role of the Na_v_1.7 channel in neuropathic pain using nociceptor-specific deletion of Na_v_1.7 in mouse. In addition, we readdressed the role of Na_v_1.8 in neuropathic pain by generating a double knockout of Na_v_1.8 and Na_v_1.7. We reasoned that the co-deletion of Na_v_1.7 in Na_v_1.8-expressing neurons should reveal any potential role for Na_v_1.8 in neuropathic pain.

## Results

### Generation of Na_v_1.7 and Na_v_1.8 double knockouts in nociceptors

Nociceptor-specific Na_v_1.7 knockout mice and their littermate controls were generated as described previously [13]. It is not possible to generate global knockouts of both Na_v_1.8 and Na_v_1.7 since global deletion of Na_v_1.7 is lethal at P0 [13]. Therefore, we generated Na_v_1.7 and Na_v_1.8 double-knockout (DKO) mice by exploiting the fact that the Na_v_1.8Cre allele has Cre sequence inserted in exon 1 followed by transcriptional stop signals [19]. Consequently homozygous Na_v_1.8Cre mice are Na_v_1.8 global-knockouts (Na_v_18 KO) and show no Na_v_1.8 currents in sensory neurons [19].

**Figure 1:**
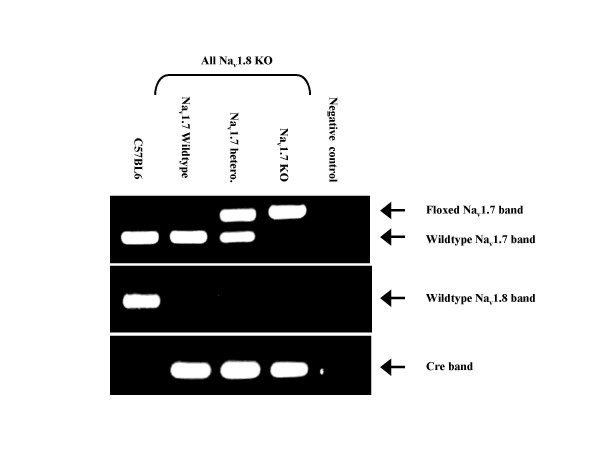
**Generation and genotyping of Nav1.7 and Nav1.8 double knockout mice. **PCR genotyping of mice generated by breeding homozygous Nav1.8Cre/heterozygous floxed Nav1.7 mice with each other (lanes 2, 3, and 4). All the three groups are Nav1.8 Knockouts, i.e. positive for the Cre band (249 bp) and negative for the wildtype Nav1.8 band (460 bp). Mice homozygous for the floxed Nav1.7 allele (461 vs. 317 bp band) are nociceptor-specific Nav1.7 knockout as well (lane 4). C57BL6 wildtype control is shown in lane 1 and no DNA negative control is in lane 5.

We compared the DKO strain to the Na_v_1.8 KO (homozygous Na_v_1.8Cre). To obtain the desired strains we mated mice homozygous for the Na_v_1.8Cre/heterozygous for floxed Na_v_1.7 allele with each other. The resulting progeny are all Na_v_1.8 Knockout. However, 25% will have wildtype Na_v_1.7 alleles, 50% will be heterozygous for the floxed Na_v_1.7 allele and 25% will be homozygous for the floxed Na_v_1.7 allele (i.e. DKO) figure 1.

Eight weeks old C57BL6 inbred mice were used as wildtype (WT) control (wildtype Na_v_1.8 and Na_v_1.7) because it would be impractical and undesirable to generate them as littermates to the test groups (DKO and Na_v_1.8 KO). The expected ratio of each of desired strains would be only 6.25% if the parents were heterozygous for both Na_v_1.8Cre and floxed Na_v_1.7 alleles compared with 25% as in our breeding strategy. The Na_v_1.8 KO/Na_v_1.7 heterozygous group was analysed in behavioural tests and was found to be similar to the Na_v_1.8 KO group (data not shown).

### Development and motor coordination

**Figure 2:**
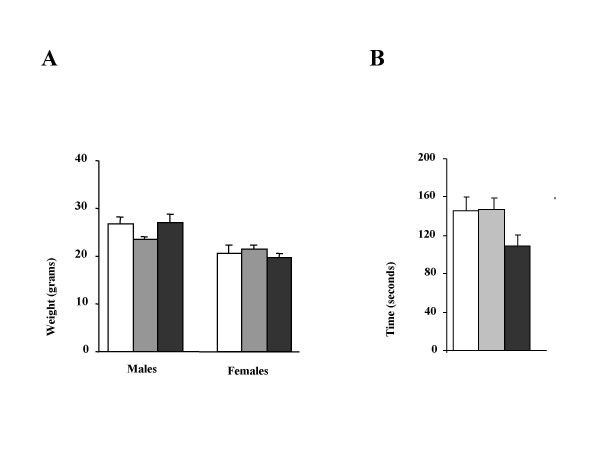
**Development and motor coordination. A**) Males and females mice have similar weights among the three groups being compared. In males the *P *value for WT vs. Nav1.8 KO is 0.09, for WT vs. DKO is 0.89 and for Nav1.8 KO vs. DKO is 0.08. In females the *P *value for WT vs. Nav1.8 KO is 0.27, for WT vs. DKO is 0.71 and for Nav1.8 KO vs. DKO is 0.27. **B**) Performance on the rotarod apparatus is not significantly different between the three mice groups. The *P *value for WT vs. Nav1.8 KO is 0.98, for WT vs. DKO is 0.38 and for Nav1.8 KO vs. DKO is 0.39. *P *values were calculated using two-tailed T-test. WT in white, Nav1.8 KO in grey and DKO in black.

The weight of male (WT 26.72 ± 1.60 gram, n = 6; Na_v_1.8 KO 23.44 ± 0.76, n = 9 and DKO 27.05 ± 1.63, n = 9) and female (WT 20.50 ± 1.77 gram, n = 3; Na_v_1.8 KO 21.43 ± 0.95, n= 3 and DKO 19.66 ± 1.03, n = 4) mice in each group was very similar and was not significantly different, figure 2a. Motor coordination as tested through performance on the rotarod apparatus was unchanged between the mouse groups studied. The time spent on the rotarod was not significantly different between the three mouse groups, figure 2b (WT 145.6 ± 13.12 sec, n = 4; Na_v_1.8 KO 146.4 ± 11.37, n = 7 and DKO 108.6 ± 11.5, n = 7).

### Acute pain thresholds

**Figure 3:**
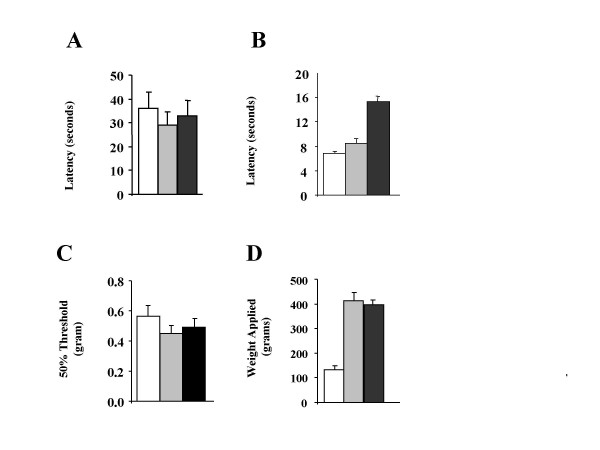
**Acute pain thresholds are increased in the DKO mice. A**) Latency to respond in the hotplate test was not different between all groups. The *P *value for WT vs. Nav1.8 KO is 0.67, for WT vs. DKO is 0.72 and for Nav1.8 KO vs. DKO is 0.43. **B**) Latency to paw withdrawal in the Hargreave's test was doubled in the DKO mice. The *P *value for WT vs. Nav1.8 KO is 0.08, for WT vs. DKO is <0.0001 and for Nav1.8 KO vs. DKO is <0.0001. **C**) The 50% withdrawal threshold to stimulation with von Frey hairs was not different between all groups. The *P *value for WT vs. Nav1.8 KO is 0.21, for WT vs. DKO is 0.25 and for Nav1.8 KO vs. DKO is 0.84. **D**) The Nav1.8 KO and DKO mice showed profound analgesia to noxious mechanical pressure applied to the tail using the Randall-Selitto apparatus. The *P *value for WT vs. Nav1.8 KO is <0.0001, for WT vs. DKO is <0.0001 and for Nav1.8 KO vs. DKO is 0.67. *P *values were calculated using two-tailed T-test. WT in white, Nav1.8 KO in grey and DKO in black.

We measured thermal pain thresholds using the hotplate and Hargreave's tests. In the hotplate test, which involves supraspinal activity, the response latency was not different between the three mouse groups (WT 36.1 ± 6.5 sec, n = 7; Na_v_1.8 KO 29.0 ± 5.6, n = 7 and DKO 32.7 ± 6.35, n = 7), figure 3a. However, the response latency in the Hargreave's test was doubled in DKO group (15.30 ± 0.91 sec, n = 7) compared to both WT (6.88 ± 0.28, n = 11) and Na_v_1.8 KO (8.47 ± 0.78, n = 7) groups, figure 3b. The latency in the Na_v_1.8 KO was higher that that of the WT group which is in agreement with the finding of Akopian *et al *using the conventional Na_v_1.8 global knockout [12]. The nociceptor-specific Nav1.7 knockout alone shows a 40% increase in latency in the Hargreave's test [13]

Pain thresholds to punctate mechanical stimulation was measured using calibrated von Frey hairs according to the up and down method [20]. The 50% withdrawal threshold was not different between the three groups (WT 0.56 ± 0.07 gram, n = 11; Na_v_1.8 KO 0.45 ± 0.05, n = 13 and DKO 0.49 ± 0.06, n = 17), figure 3c. In contrast pain threshold to noxious mechanical pressure applied to the tail using the Randall-Selitto apparatus was much higher in both the Na_v_1.8 KO (412.4 ± 33.97 gram, n = 7) and DKO (395.7 ± 21.03, n = 7) groups compared to that of the WT (131.4 ± 16.54, n = 6) group, figure 3d. Usually the cut off point (500 gram) was reached without an observed escape response in the Na_v_1.8 KO and DKO groups, indicating a high resistance to static blunt mechanical pressure. There was no difference between the Na_v_1.8 and the DKO mice in this test, figure 3d, and similar results have been obtained with the nociceptor-specific Na_v_1.7 knockout [13]

### Inflammatory pain behaviour

**Figure 4:**
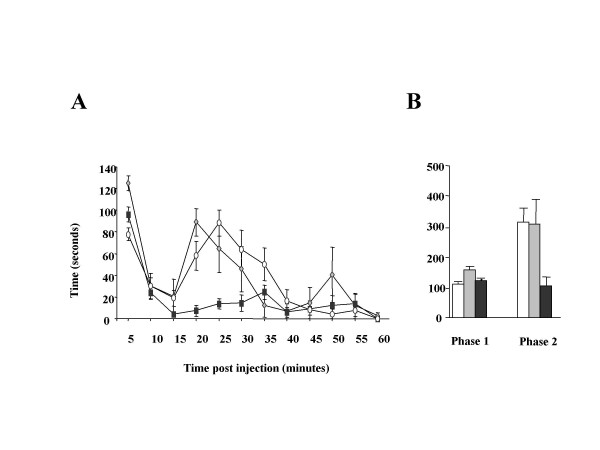
**Reduced pain behavior in formalin test in DKO. A**) The pain response after injection of 5% formalin in hindpaw showed the typical biphasic course. **B**) The second phase of the formalin response was reduced to 30% in the DKO mice compared to WT and Nav1.8 KO mice. The *P *value for WT vs. Nav1.8 KO is 0.94, for WT vs. DKO is 0.001 and for Nav1.8 KO vs. DKO is 0.08. The first phase was not significantly different between all groups. The *P *value for WT vs. Nav1.8 KO is 0.60, for WT vs. DKO is 0.30 and for Nav1.8 KO vs. DKO is 0.96. *P *values were calculated using two-tailed T-test. WT in open circles, Nav1.8 KO in grey triangles and DKO in black squares.

The pain response elicited by injection of 20μl of 5% formalin intradermally in the hindpaw showed the typical biphasic response in the three groups, figure 4a. The first phase (1–10 minutes) was not different between the three groups (WT 107.8 ± 10.12 sec n = 8, Na_v_1.8 KO 155 ± 11.00 n = 4, DKO 120.4 ± 5.96 n = 8), figure 4b. In contrast, the second phase (10–60 minutes) was much reduced in the DKO group (105.4 ± 28.43 sec) compared to both the Na_v_1.8 KO (309 ± 80.85, *P *= 0.08) and the WT (216 ± 43.67, *P *= 0.0016) groups, figure 4b. This effect was even more dramatic than that observed in the nociceptor specific Nav1.7 knockout [13] We did not study other inflammatory pain models since the nociceptor-specific Na_v_1.7 knockout mouse is completely deficient in commonly used inflammatory pain models [13]. It would be impossible therefore to measure a further reduction in inflammatory pain behaviour in the DKO.

### Neuropathic pain in nociceptor-specific Na_v_1.7 knockout mice

**Figure 5:**
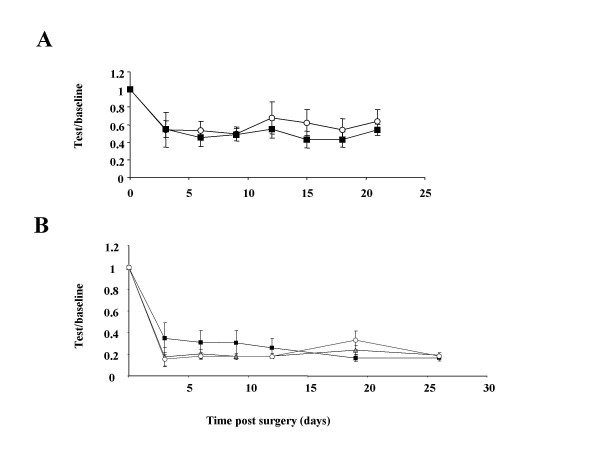
**Analysis of neuropathic pain in nociceptor-specific Nav1.7 knockout mice and double knockout mice. **(**A**) Both control (white circles, mean 0.56 ± 0.9, n = 8) and nociceptor-specific Nav1.7 KO (black squares, 0.48 ± 0.8, n = 11) developed robust mechanical allodynia after ligation of spinal nerve L5. There is no difference in the extent of pain behavior at any time point (*P *= 0.49 ANOVA). (**B**) WT (open circles), Nav1.8 KO (grey triangles) and DKO (black squares) developed profound mechanical allodynia after ligation of spinal nerve L5.

To study neuropathic pain behavior we induced peripheral nerve injury using to the Chung model (ligation of L5 spinal nerve) in the Na_v_1.7 nociceptor-specific knockout and homozygous floxed-Na_v_1.7 as controls. Both groups developed a robust mechanical allodynia starting form the third day post surgery, figure 5a. The extent and time course of development of increased mechanosensitivity was identical in both nociceptor-specific Nav1.7 knockout and their littermate controls.

### Neuropathic pain in Nav1.7 Nav1.8 double knockout mice

To address the issue of compensatory up-regulation of Na_v_1.7 in Na_v_1.8 knockout we induced peripheral nerve injury in WT, Na_v_1.8 KO and DKO. All mice groups studied developed a robust mechanical allodynia starting form the third day post surgery, figure 5b. There were no statistically meaningful differences in the behaviour of the groups of mice.

## Discussion

This study confirms that the two sodium channel α- subunits Na_v_1.7 and Na_v_1.8, expressed selectively in nociceptive sensory neurons, have important roles in nociception and in pain. In the absence of specific pharmacological blockers, the use of genetic approaches, a combination of global and nociceptor-specific knockouts, has enabled us to carry out studies exploring the contribution of these two isoforms of VGSCs in different pain conditions. Na_v_1.8 knockouts are already known to have deficits in inflammatory and visceral but not neuropathic pain [12]. Na_v_1.7 nociceptor-specific knockouts are almost totally refractory to changes in peripheral pain thresholds evoked by inflammatory mediator [13]. This mirrors the inflammatory phenotype of Na_v_1.7 gain-of-function mutations in erythermalgia [21]. This heritable disorder is caused by point mutations that lead to altered thresholds of activation in Na_v_1.7 [22]

### Acute pain thresholds

analysis of acute mechanical thresholds in DKO mice confirmed previous findings that the knockout of either Na_v_1.8 or Na_v_1.7 renders the mice resistant in the Randel-Selitto test of noxious mechanical pressure while the responses to von Frey hairs remain unchanged compared to controls [12,13]. Furthermore, it has been shown that both Na_v_1.8 and nociceptor-specific Na_v_1.7 knockouts have small increases of about 20–40% in their thermal threshold [12,13]. Interestingly, we found that the thermal threshold in the Hargreave's test was doubled in the DKO compared to Na_v_1.8 knockout or WT controls, figure 3b. The increase is more than the sum of the phenotype in the individual knockouts and may indicate that Na_v_1.8 and Na_v_1.7 are the major VGSC isoforms present in nociceptive terminals and their deletion dramatically increases pain threshold. This is supported by the fact that Na_v_1.7 is transported to nerve terminals of sensory neurons [10] and that Na_v_1.8 currents are localized in the terminals [23].

### Inflammatory pain

We have not studied pain behavior in inflammatory models where the nociceptive-specific Na_v_1.7 knockout has shown complete deficits in inflammatory pain behavior [13], because it would not be possible to observe further changes brought about by the co-deletion of Na_v_1.8. We only investigated the pain response in the formalin model. While the nociceptive-specific Na_v_1.7 knockout showed a reduction in the second phase to about 50% of controls [13] the DKO showed a slightly bigger reduction to 30% of controls, figure 4b. This is in contrast to Nav1.8 mice that showed no reduction in the second phase compared to controls, figure 4b. Therefore, the reduction in the second phase in the DKO is a new phenotype rather than a mere summation of the phenotype in the individual knockouts. This highlights further the predominant role for Na_v_1.7 in inflammatory pain. Expression of Na_v_1.7 has been reported to increase in sensory neurons subsequent to induction of inflammation [24].

This study and our previous [13] work have explored the role of Na_v_1.7 in Na_v_1.8-expressing neurons, since our strategy exploits the Na_v_1.8 promoter to drive Cre expression. We have shown previously using non-quantitative RT-PCR that not all the Na_v_1.7 mRNA in DRGs from the nociceptor-specific Na_v_1.7 knockout is deleted [13]. This indicates that there is a population of sensory neurons, the size of which has yet to be determined, that express Na_v_1.7 but not Na_v_1.8. Therefore, the role of Na_v_1.7 in sensory neurons where Na_v_1.8 is not expressed (and which do not express Cre) remains to be studied.

From all the above, our results indicate that Na_v_1.8 and Na_v_1.7 subunits contribute to the excitability of peripheral nerve terminals and their modulation is critical for peripheral sensitisation in inflammatory pain

### Neuropathic pain

We investigated the role of Na_v_1.7 expressed in nociceptors in neuropathic pain. Analysis of neuropathic pain in the nociceptor-specific Na_v_1.7 knockout using the Chung model showed that they developed mechanical allodynia to the same extent as the littermate control mice (homozygous floxed Na_v_1.7), figure 5. This clearly shows that unlike its critical role in inflammatory pain [13], Na_v_1.7 does not contribute to neuropathic pain.

In addition, the Na_v_1.8 knockout and WT as well as the DKO mice developed a profound mechanical allodynia the level of which was indistinguishable between the three groups. This provides further evidence that Na_v_1.8 does not contribute to mechanical allodynia as reported by Kerr *et al *[17]. In addition our results rule out Na_v_1.7 up-regulation in the Na_v_1.8 knockout as the reason behind the discrepancy between the finding of Kerr *et al *[17] and Lai *et al *[18]. Surprisingly Lai et al [18] failed to detect the changes in acute pain thresholds observed in the Na_v_1.8 KO both in this study and that of Akopian *et al *[12], which would be expected to occur if Na_v_1.8 had been down-regulated.

The lack of changes in mechanical allodynia in any individual or even in double knockouts of Na_v_1.8 and Na_v_1.7 subunits is consistent with recent data by Flake *et al*. They reported that changes in sodium currents shortly after nerve injury do not correlate with an increase in neuronal excitability and that the sum of changes to ionic currents, and not a single class of voltage-gated ion channel, underlie increased neuronal excitability [25]. This does not, however, exclude a potential role for both Na_v_1.7 and Na_v_1.8 in spontaneous neuropathic pain as suggested by the finding that neuromas in the Na_v_1.8 knockout mouse display less ectopic discharges than wildtype littermates [26]. These studies confirm the importance of Na_v_1.7 and Na_v_1.8 in the physiological processes that underlie altered peripheral thresholds in inflammatory pain. Neuropathic pain that arises as a consequence of nerve damage and neuronal dysfunction is, however, not dependent on the presence of these two sodium channels. In terms of the contribution of other α-subunits to neuropathic pain, a strong correlation has been found between the expression of Na_v_1.3 and the appearance of neuropathic pain. GDNF that reverses neuropathic pain behaviour also normalises Na_v_1.3 expression [27], and recent antisense studies have supported the view that increased Na_v_1.3 expression contributes to neuropathic pain development [28]

## Conclusion

In summary, our results indicate a critical role for Na_v_1.7 and Na_v_1.8 in setting pain thresholds of nociceptive nerve terminals and in their sensitisation following tissue damage and inflammation. However, the presence of these channels, or changes to their expression and function are not required for the establishment of mechanical allodynia arising from nerve injury.

## Methods

### Genotyping of mice strains

Na_v_1.8Cre and floxed Na_v_1.7 lines were produced as described [13]. Both strains were back-crossed at last 5 times onto a C57/BL6 background.

Genomic DNA was prepared from tail biopsies and genotyped using PCR. Primers for Na_v_1.7 are (CAGAGATTTCTGCATTAGAATTTGTTC) and (AGTCTTTGTGGCACACGTTACCTC) which give a WT band of 317 bp and a floxed band of 461 bp. Primers for Na_v_1.8 are (TGTAGATGGACTGCAGAGGATGGA) and (ttacccggtgtgtgctgtagaaag) which give a WT band of 460 bp. Na_v_1.8Cre was detected by primers (aaatttgcctgcattaccggtcga) and (aaatgttgctggatagtttttactgcc) located inside Cre sequence which give a band of 249 bp.

### Behavioural analysis

All tests were approved by the United Kingdom Home Office Animals (Scientific Procedures) Act 1986 and performed in a Home Office designated room at 22 ± 2°C. Experiments were performed on animals of at least 8 weeks of age. Behavioral tests were done as before [19]

## Competing interests

The author(s) declare that they have no competing interests.

## Authors' contributions

MAN generated the mice, performed behavioral tests and wrote the manuscript.

AL carried out the neuropathic pain study of the DKO and Nav1.8 KO.

LCS Analysed the Nav1.8Cre mouse.

JNW: supervised experiments and corrected the manuscript.

All authors read and approved the final manuscript
